# Hemophagocytic Lymphohistiocytosis Secondary to Disseminated Histoplasmosis in HIV Seronegative Patients: A Case Report and Review of the Literature

**DOI:** 10.3389/fcimb.2022.847950

**Published:** 2022-06-16

**Authors:** Hongchao Chen, Qing Yuan, Hangbin Hu, Jie Wang, Meihong Yu, Qing Yang, Tingting Qu

**Affiliations:** ^1^ Deparment of Laboratory Medicine, The First Affiliated Hospital, Zhejiang University School of Medicine, Hangzhou, China; ^2^ State Key Laboratory for Diagnosis and Treatment of Infectious Diseases, Collaborative Innovation Center for Diagnosis and Treatment of Infectious Diseases, National Clinical Research Center for Infectious Diseases, Zhejiang University School of Medicine First Affiliated Hospital, Hangzhou, China

**Keywords:** *Histoplasma capsulatum*, histoplasmosis, hemophagocytic lymphohistiocytosis, HIV seronegativity, immunocompetence, liposomal amphotericin B

## Abstract

Hemophagocytic lymphohistiocytosis (HLH) secondary to *Histoplasma capsulatum* infection is a rare disorder with poor outcome. Although cases of patients with human immunodeficiency virus (HIV) infection have been well documented, little study has reported in the setting of HIV seronegative. In this study, we report a case of HLH secondary to histoplasmosis in an immunocompetent patient in China and review all cases on this situation. The objective was to summary their epidemiology, clinical characteristics, diagnostic approaches, and therapeutic response. A 46-year-old male cooker presented fever, fatigue, anorexia, and weight loss. Bone marrow examination suggest fungus organism and hemophagocytosis, and further, bone marrow culture confirmed *Histoplasma capsulatum*, as the etiology of HLH. The patient was successfully treated. We reviewed a total of the 13 cases (including our patient) of HLH with histoplasmosis in intact immunology patients. Twelve of the 13 patients are from endemic areas, and nine of the 12 cases are from emerging endemic areas, India and China. Three patients had sojourn history may related to the disease onset. Twelve of the 13 cases fulfilled HLH-2004 criteria. The diagnosis of *Histoplasma capsulatum* infection was established by histological examination (13 of 13), culture (4 of 13), molecular method (2 of 13), and antigen or serological assays (2 of 13). Amphotericin B, posaconazole, and itraconazole show favorable activity against the fungus, seven patients used specific treatment for HLH. For analysis of outcomes, two of the 13 patients died. Our present case report and literature review show that disseminated *Histoplasma capsulatum* infection with HLH in the immunocompetent population becomes increasingly common in emerging endemic areas and have high mortality. It is necessary for clinicians to improve the awareness of disease diagnosis due to the atypical population and disease presentation. Timely diagnosis and early use of antifungal agents will lead to favorable prognosis.

## Introduction

*Histoplasma capsulatum* is an environmental dimorphic fungus that causes histoplasmosis (Scheel et al., 2014). With a worldwide incidence, epidemic distribution mainly in North America and Latin American countries (Matos Baltazar et al., 2016). Many areas of Asia, including India, Southeast Asia, and China along Yangtze River, are also endemic (Wheat et al., 2016; Azar et al., 2020). Infection occurs when inhalation of fungal spores or hyphae from the environment soil, and the lung is the primary organ of infection. *H. capsulatum* can disseminate throughout the body in immunocompromised persons (Crossley et al., 2016; Matos Baltazar et al., 2016), who are at least 10 times more likely to develop progressive disseminated histoplasmosis (PDH) than the general individuals (Azar et al., 2020), without timely diagnosis and treatment can lead to fatal illness. In immunocompetent and non-respiratory patients, they are usually clinically silent or mild manifestations, and diagnosis is often so challenging that the rate of underdiagnosis is high.

Hemophagocytic lymphohistiocytosis (HLH) is a rare clinical disorder, charactered by unbridled and persistent immune activation of natural killer (NK), cytotoxic T cell. Failure to control the immune response leads to injury of multiple organs (Solomon et al., 2018). It can be divided into familial and acquired. Familial hemophagocytic lymphohistiocytosis (FHL) has a fundamental property of autosomal recessive inheritance, which is typically onset in infancy period (Bryceson et al., 2007). Acquired HLH is association with systemic infections, malignant diseases, or autoimmune disorders, cases onset mainly in adults (Al-Samkari and Berliner, 2018). Most cases of HLH secondary to *Histoplasma capsulatum* infection present in immunocompromised patients (Gil-Brusola1 et al., 2007; Nguyen et al., 2020; Castejón-Hernández et al., 2021), and clinicians are vigilant in diagnosing the disease in endemic areas.

Here, we present a rare case of an immunocompetent cooker with *Histoplasma* infection and secondary HLH, which was successfully treated by immunoglobulin and high-dose prednisone immunosuppression combined with antifungal therapy. A review of literature on this situation is also performed.

## Case Presentation

In late August 2020, a 46-year-old male was admitted to our emergency department, with complaints of intermittent fever associated with fatigue and anorexia lasting for a month. He also complained of 30-kg weight loss within 5 months. The patient first visited a local hospital 2 weeks ago. Because of fever and lymphadenopathy, a bone marrow aspiration was performed. The bone marrow smear revealed hemophagocytosis and yeast-like bacteria; fungal infection was considered. However, no fungus was cultured in the bone marrow culture. Therefore, empirical antifungal treatment of Voriconazole was prescribed. After taking the drug, the patient developed insomnia and was replaced by itraconazole, without symptom improvement. He works as a cooker and gets up at 1 a.m. every day. He contacted with a soil-based environment for building and construction around his house. He lived in a rural area in Southeast China for more than 40 years and never traveled to foreign countries.

At physical examination, the patient has temperature of 39.7°C, pulse rate of 129/min, respiratory rate of 18/min, and blood pressure of 119/63mm of hg and has normal oxygen saturation. He had pallor and hepatosplenomegaly. Heart and lung auscultation were unremarkable.

The laboratory examination indicated pancytopenia at admission: hemoglobin, 63 g/L; platelet, 44 × 10^9^/L; leucocytes, 1.6 × 10^9^/L; neutrophils, 1.3 × 10^9^/L. The C-reactive protein (CRP) level was 44.4 (normal, < 8) mg/L, and procalcitonin (PCT) level was 1.65 (normal, <0.15) ng/ml. Fibrinogen levels was decreased (1.0g/L). Serum ferritin was markedly elevated (2,775.0 ng/ml). The CD4 cell count was 120 cells/μl. He had mild abnormal serum levels of aspartate aminotransferase (AST) with 81U/L, alanine aminotransferase (ALT) with 53 U/L, and glutamyl transpeptidase (GGT) with 118 U/L, respectively. There was a decline in albumin levels, 32.0 g/L. The kidney function markers were within normal ranges. The patient had a detectable level of (1 → 3)-β-d-glucan, 212.56 (normal, < 60) pg/ml, and the GM (galactomannan) level was not elevated. Serological results for human immunodeficiency virus (HIV), Epstein–Barr virus, cytomegalovirus, hepatitis virus B and C, COVID-19, and the T-cell spot test for tuberculosis infection (T-SPOT) were negative. His blood culture did not grow any pathogenic organism. **Table 1** shows the results of relevant blood investigations.

**Table 1:** Results of relevant blood investigations.

Test	Result
Autoimmunological antibodies	Negative
Leucocytes	1.6 × 10E9/L (normal, 4–10)
Hemoglobin	63 g/L (normal, 131–172)
Platelet	44 × 10E9/L (normal, 83–303)
C-reactive protein	44.4 mg/L (normal, <8)
Procalcitonin Fibrinogen	1.65 ng/ml (normal, <0.15) 1.0 g/L (normal, 2-4)
Hepatic function test results	Mild abnormal
Prothrombin time(PT)	13.7 s (normal, 10-13.5)
D-dimer	2,451 µg/L (normal, <700)
Serum ferritin	2,775.0 ng/ml (normal, <323)
Renal function test results	Within normal range
Immunoglobulin A	77.0 mg/dl (normal, 100–420)
Immunoglobulin M	14.0 mg/dl (normal, 30–220)
Immunoglobulin G and complement proteins C3 and C4	Within normal range
Blood culture	Negative

**Figure 1:**
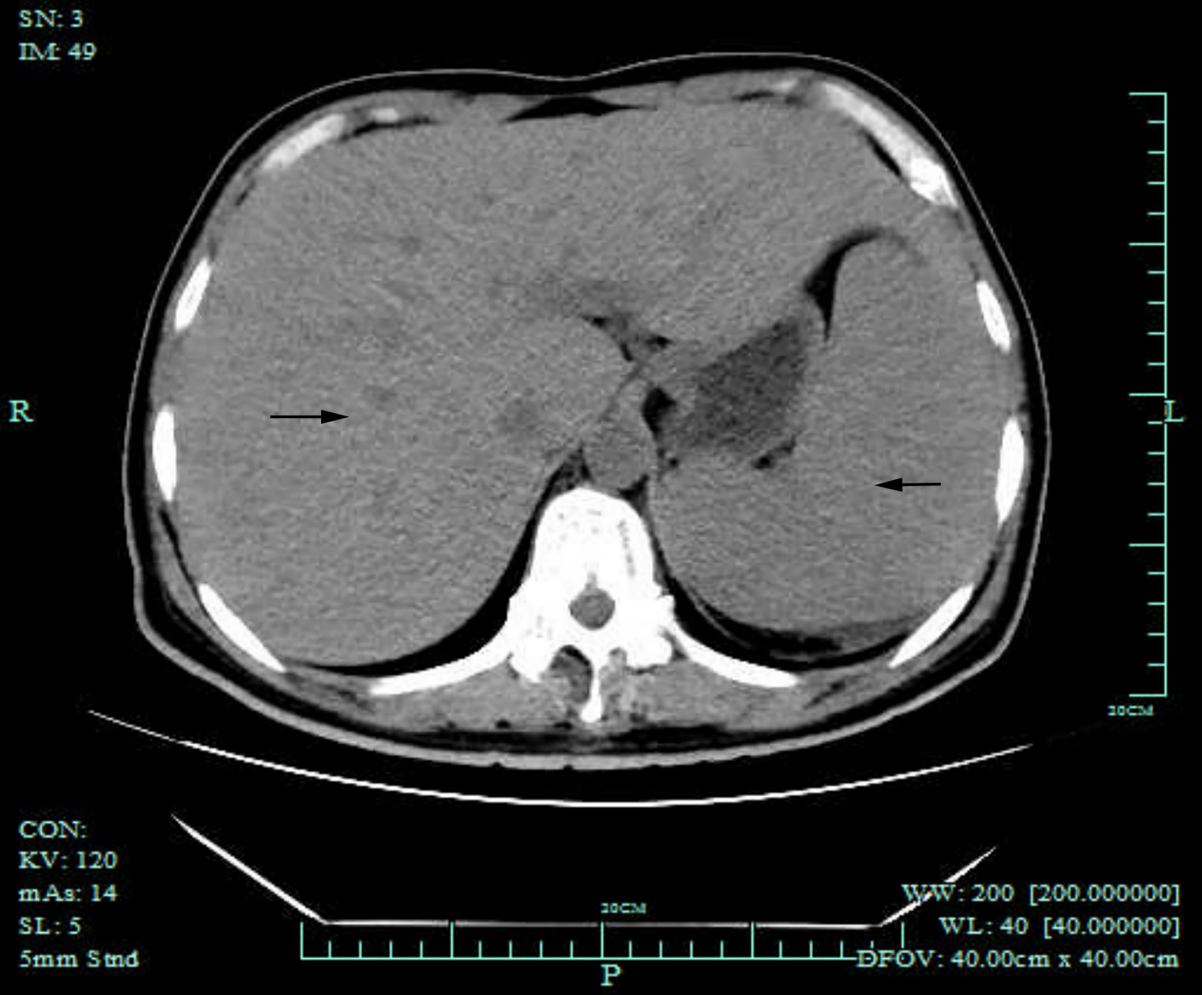
Abdominal CT scan showed splenomegaly and hepatomegaly (black arrows).

**Figure 2:**
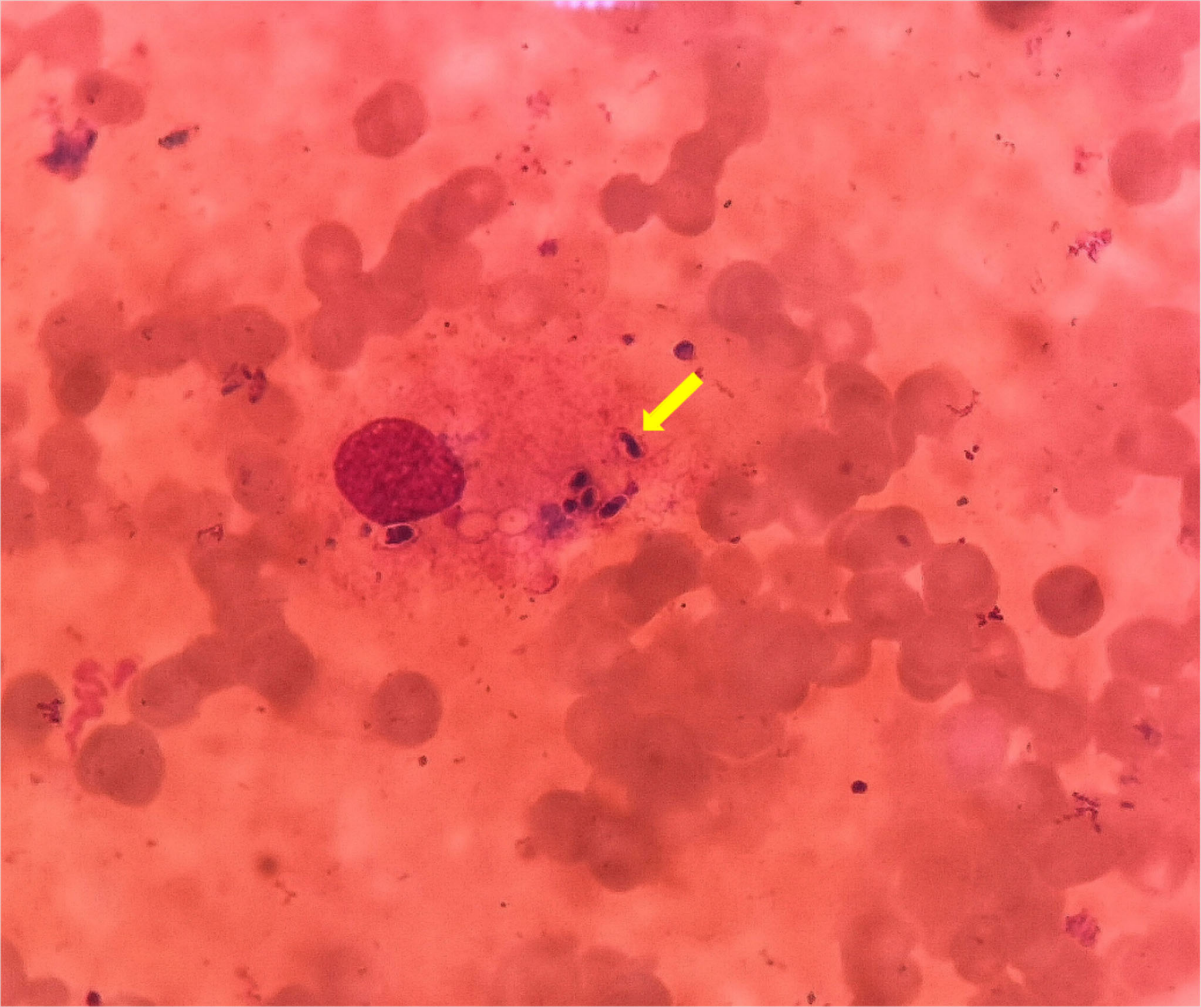
HE stain of bone marrow puncture images (×1,000). These oval or round organisms with amaranth nuclei and capsule-like unstained halos around observed in the cytoplasm of phagocytes (yellow arrow).

**Figure 3:**
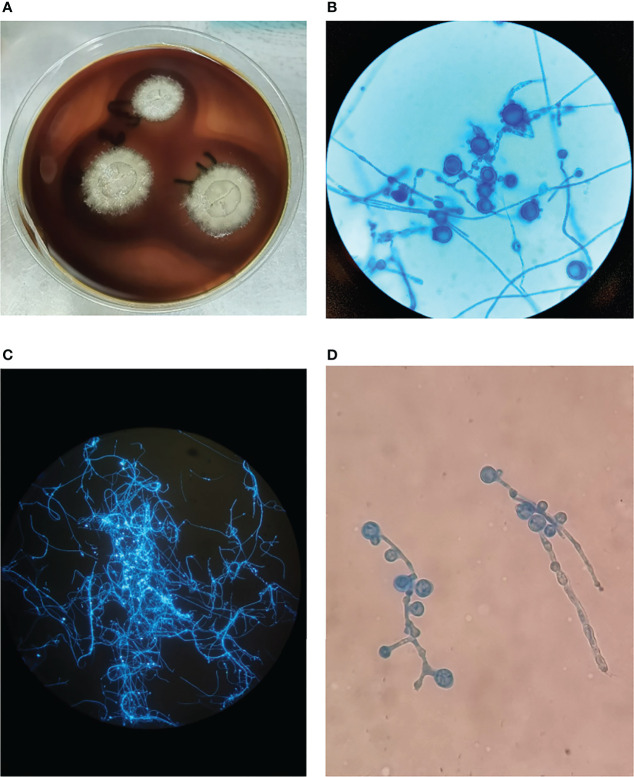
**(A)** The colonies of *Histoplasma capsulatum* on Columbia blood agar medium after subculture, the fungus is milky white with villous hyphae in the outer ring. **(B)** Cogwheel-like macroconidia of *Histoplasma capsulatum* stained with Lactophenol cotton blue after subculture, ×400 magnification. **(C)** Hyphae and spores are bright blue fluorescent in Fluorescence staining, 100× magnification. **(D)** Lactophenol cotton blue staining in primary culture with cogwheel-like macroconidia, ×400 magnification.

Computed tomography (CT) of the chest was normal but the abdominal CT showed hepatosplenomegaly and posterior peritoneum lymphadenopathy (**Figure 1**). PET/CT revealed hepatosplenomegaly and posterior peritoneum lymphadenopathy and bone marrow with high FDG uptake, suggesting lymphoma probably. A bone marrow puncture was done at admission which displayed hemophagocytosis and negative result for bacterial culture. Hematoxylin and eosin (HE)–stained bone marrow demonstrated oval or round organisms with amaranth nuclei and capsule-like unstained halos around these organisms observed in the cytoplasm of phagocytes (**Figure 2**). The fungus culture of bone marrow was also sent to laboratory for further identification. After 8 weeks of initial plating on potato dextrose agar (PDA) medium at 25°C and subsequently sub-cultured at intervals of 2 weeks in Columbia blood agar medium at 25°C. These are shown in **Figure 3**, which finally confirmed as *Histoplasma capsulatum*.

The patient received empirical treatment with oral itraconazole from the day of admission (200 mg three times daily for 7 days). At day 2 after admission, the body temperature was back to normal. The laboratory examination were repeated at day 4 after admission: hemoglobin, 69 g/L; platelet, 67 × 10^9^/L; leucocytes, 4.7 × 10^9^/L, neutrophils: 4.2 × 10^9^/L. Serum ferritin declined to 1,264.3 ng/ml. After the results of the bone marrow puncture come out, liposomal amphotericin B was prescribed (1.0 mg/kg daily for 10 days), followed by oral itraconazole (200 mg twice daily for a total of at least 12 months). For HLH, the patient received treatment with prednisone 40 mg daily and intravenous immunoglobulin 20 g daily for 5 days. The blood cell count, CRP, PCT, and serum ferritin improved substantially after a week. The CD4 cell count also recovered normal after a week.

In this case, the patient presented with HLH with fever, splenomegaly, elevated ferritin, hypofibrinogenemia, blood cell abnormalities, and hemophagocytosis. Although he had no immunocompromised disease and never went to epidemic area, but he had been short of sleeping time for long time, and there was a lot of dust around his house because of building a road. It is unclear whether this may have been a risk factor for disease.

## Literature Review

Case report and case series for HLH and histoplasmosis were identified in the five databases (Pubmed, Embase, Web of science, China National Knowledge Infrastructure, and Wanfang Data) by using search criteria (“lymphohistiocytosis, hemophagocytic” [MeSH Terms] AND “Histoplasmosis” [MeSH Terms]). The case being reported here is also included in the analysis and review of literature. The following criteria were used for the inclusion: 1) fulfilled HLH 2004 criteria or have a definition of HLH; 2) the infection of *Histoplasma capsulatum* is confirmed by standard methods; 3) immunity is normal, excluding diseases with compromised immunity, immunosuppressive medications therapy, and autoimmune diseases. In the past, clinicians’ understanding of HLH was relatively insufficient, so it cannot be ruled out that we have missed some literature on HLH secondary to *H. capsulatum* infections.

A total of 90 articles were retrieved from the databases without time limit, including 77 papers in English, 8 in Spanish, and 2 in Chinese. After discarding three articles for incomplete information, the remaining 87 literature contain a total of 128 patients. This review of literature identified 12 articles with 12 cases (9.4%), presenting a confirmed HLH with histoplasmosis of intact immunology. For the excluded articles, 65 of 128 (50.8%) patients were infected with HIV; 20 of 128 (15.6%) had rheumatic disease; 15 of 128 (11.7%) had organ transplant history (14 cases with renal transplant, and one case with heart transplant); and 3 of 128 (2.3%) had hematologic malignancy. Immunosuppressive medications therapy containing tumor necrosis factor inhibitors, steroid hormone, and chemotherapy were used in 43 of 128 (33.6%) patients. Details are displayed in the **Supplementary Materials**.

The features of the 12 patients and our case with competent immunology are summarized in the **Table 2**. Two of them were infants, and the others were all older than 16 years. Among the 13 cases whose sex was mentioned, 10 were male patients, including our patient. Five patients were China born (including our patient), two of them had a sojourn history in Africa, four patients were India born, three patients were America born, and one patient was Germany born but had a visiting of a bat cave when she had traveling in Thailand 8 months before onset. Three patients had job as cooker, blood bank worker, and homemaker; occupations of other seven patients were unknown.

**Table 2:** Case reports on HLH due to histoplasmosis in HIV seronegative patients.

Reference	(Kashif et al., 2015)	(Mukherjee and Basu, 2015)	(Sonavane et al., 2016)	(Ferguson-Paul et al., 2016)	(Schulze et al., 2017)	(Bommanan et al., 2017)	
Published year	2015	2015	2016	2016	2017	2017	
Number of cases	1	1	1	1	1	1	
Home country	USA	India	India	USA	Germany	India	
Age/Gender	34y/M	52y/M	43y/F	6m/F	59y/F	32y/M	
Occupation	/	/	homemaker	NA	/	/	
History of sojourn	Nigeria	/	/	–	Thailand/ Costa Rica	–	
Clinical manifestation							
Fever	+	+	+	+	+	+	
Fatigue	–	–	+	–	–	–	
Rash	–	+	–	–	–	–	
Weight loss	+	–	+	–	+	+	
Anorexia	+	–	+	–	–	–	
Cough	–	–	+	–	–	–	
Foot edema	–	+	+	–	–	–	
Co-infections	–	–	M.TB	–	EBV/Hafnia alvei	–	
Underlying disease	Sickle cell disease	COPD/DM/EN	TB	–	breast cancer	–	
Leucocytes (k/μl)	10	4.2	2.6	/	12.37	1.4	
Neutrophils (k/μl)	/	/	/	1.5	/	1.2	
Hemoglobin (g/dl)	4.9	6	7	5.9	5.8	7.9	
Platelet (k/μl)	48	/	91	11	128	40	
Triglycerides (mg/dl)	135	/	NR	378	270	/	
fibrinogen (g/L)	0.69	/	0.53	0.78	/	/	
NK cell activity	/	/	/	NR	/	/	
Serum ferritin (μg/L)	7,493	3.06	891.7	1,218	99,919	3,339	
Soluble CD25 (U/ml)	/	/	/	21,530	4,445	/	
ALT (U/L)	5,058	/	23	/	44	62	
AST (U/L)	16,637	/	28	/	39	52	
LDH	1,466	/	296	/	306	/	
Hepatomegaly	+	+	+	+	–	+	
Splenomegaly	+	+	+	+	–	+	
Lymphadenopathy	+	+	–	–	+	–	
Pulmonary disorders	+	/	+	+	+	–	
Number of HLH-2004 criteria	6	3	6	7	5	5	
Hemophagocytosis	BM Bx	BM Bx	BM Path	BM Path	BM Path	BM Path	
Diagnosis of histoplasmosis							
Path	LN Bx	liver/spleen Bx	BM Path	BM Path	colon/liver/LN/lung Bx	BM Path	
Culture	/	/	/	/	/	/	
Antigen assays	–	/	/	Serum/urine/CSF	/	/	
Antibody assays	/	/	/	+	/	/	
Molecular method	/	/	/	/	+	/	
Antifungal treatment	AmB/Itra	AmB	AmB/Itra	AmB/Itra	AmB/Posa	AmB	
HLH-specific treatment	DE	–	–	DE	–	–	
Outcomes	Died	Died	Recovery	Recovery	Recovery	Recovery	
Reference	(Xiong et al., 2017)	(Pancoast et al., 2019)	(Gupta et al., 2019)	(L Xiaolin et al., 2021)	(Song et al., 2021)	(Sijie et al., 2022)	PS
Published year	2017	2019	2019	2020	2021	2022	–
Number of cases	1	1	1	1	1	1	1
Home country	China	USA	India	China	China	China	China
Age/Gender	37y/M	3m/M	29y/M	27y/M	16y/M	44y/M	46y/M
Occupation	blood bank worker	NA	/	/	NA	/	Cooker
History of sojourn	–	/	/	/	/	Kenya	–
Clinical manifestation							
Fever	+	+	+	+	+	+	+
Fatigue	–	–	–	+	–	+	+
Rash	–	–	–	–	–	–	–
Weight loss	+	–	+	–	–	+	+
Anorexia	–	–	+	–	–	+	+
Cough	+	–	+	–	–	+	–
Foot edema	–	–	–	–	–	+	–
Co-infections	CMV HSV	–	–	HBV	–	–	–
Underlying disease	–	–	–	Hepatitis	–	–	–
Leucocytes (k/μl)	1.86	/	3.9	3.45	2.35	2.2	1.6
Neutrophils (k/μl)	1.23	/	/	/	0.84	1.63	1.3
Hemoglobin (g/dl)	8.6	/	8.4	8.7	11.8	6.3	6.3
Platelet (k/μl)	2	/	79	32	62	4	44
Triglycerides (mg/dl)	/	213	304	/	1.44	/	NR
Fibrinogen (g/L)	/	1.32	<1.5	3.6	1.6	/	1
NK cell activity	/	/	/	/	NR	Below NR	/
Serum ferritin (μg/L)	543.3	830	>2,000	1,900	403.8	2,545	2775
Soluble CD25 (U/ml)	/	9,560	/	>7,500	>44,000	35,854	/
ALT (U/L)	82	/	/	98.6	32	40	53
AST (U/L)	70	/	/	32.3	45	49	81
LDH	352	/	579	468	/	/	122
Hepatomegaly	+	+	+	+	+	+	+
Splenomegaly	+	+	+	+	+	–	+
Lymphadenopathy	+	–	–	+	+	–	+
Pulmonary disorders	+	–	–	+	–	+	–
Number of HLH-2004 criteria	5	6	5	6	5	6	6
Hemophagocytosis	BM Path	BM Path	–	BM Path	BM Path	BM Path	BM Path
Diagnosis of histoplasmosis							
Path	BM Path	BM Path	BM Path	BM Path	BM Path	BM Path	BM Path
Culture	blood	/	/	/	BM	BM	BM
Antigen assays	/	serum/urine	/	/	/	/	/
Antibody assays	/	/	–	/	/	/	/
Molecular method	/	/	/	/	/	+	/
Antifungal treatment	AmB	AmB/Itra	AmB/Itra	AmB	AmB	AmB/Itra	AmB/Itra
HLH-specific treatment	IVIG	–	–	DEP	DEP	IVIG	IVIG/Prednisone
Outcomes	Recovery	Recovery	Recovery	Recovery	Recovery	Recovery	Recovery

PS, present study; NR, normal range; NA, not applicable; DE, dexamethasone and etoposide; DEP, liposome doxorubicin, etoposide, and methylprednisolone; IVIG, intravenous immunoglobulin; Bx, biopsy; LN, lymph node; BM, bone marrow; Path, histopathology; COPD, chronic obstructive pulmonary disease; DM, diabetes-mellitus; EN, erythema nodosum; TB, tuberculosis; CSF, cerebrospinal fluid; EBV, Epstein–Barr virus; AmB, amphotericin B; Itra, itraconazole; Posa, posaconazole.

The nonspecific clinical syndrome consists of fever (13 of 13), weight loss (8 of 13), anorexia (5 of 13), fatigue (4 of 13), and cough (4 of 13). Among 13 patients whose laboratory tests were mentioned, significant cytopenias involving at least two cell lines, elevated ferritin, hypertriglyceridemia, elevated LDH, elevated hepatobiliary enzymes, and organomegaly (hepatomegaly, splenomegaly, and lymphadenopathy) are common symptoms. NK cell activity was detected in three study, but only one patient showed low activity. High-soluble interleukin-2 receptors (CD25) were confirmed in six cases. Pulmonary disorders with cough or imaging presentation were showed in seven cases. Hemophagocytosis was visualized in bone marrow histopathology or biopsy in 12 patients. For diagnosis of HLH, except one patient with insufficient information only meet three of eight HLH-2004 criteria; the other 12 patients with PDH all fulfilled at least five of eight HLH-2004 criteria.

In the cases reviewed, histological and cytological examinations in bone marrow (10 cases) and colon/liver/lymph node/lung/spleen (three cases) lead to fast diagnosis of Histoplasma capsulatum. The fungal was culture in three cases in bone marrow and one case in blood. The etiology was identified by molecular methods in two patients. Antigen and serological assays of histoplasma capsulatum were less used in clinical practice, only detected in two patients.

All patients are treated with first line systemic antifungal formulation with liposomal amphotericin B, and eight disseminated infection patients received step down to azoles as recommended by the Infectious Diseases Society of America (Wheat et al., 2007). Seven patients used itraconazole, and one severe patient took posaconazole due to increasing *in vitro* activity. For specific treatment of HLH, four cases were initiated with immunosuppressed treatment recommended by HLH-94 protocol and clinical trials, and three cases were treated with intravenous immunoglobulin. For analysis of outcomes, 2 of the 13 (15.4%) patients died, clinical status deteriorated rapidly in 1 patient, and the other had complicated underlying disease. Condition was improved in 11 patients after antifungal and HLH-specific treatment.

## Discussion

Environmental soil is the reservoir of *Histoplasma capsulatum* (Kauffman, 2007). Contaminated soil of bird or bat cave, especially that found beside chicken coops or under blackbird roosts, provides a luxuriant condition for mold growth (Kauffman, 2007). In the cases reviewed, the tourism exposure risk to a bat cave of 1 patient who live in non-endemic countries is specified. Histoplasmosis is endemic worldwide; in our literature review, five cases were from China and four cases were from Africa, and two Chinese patients had a history of sojourn in Africa. This suggests that clinicians need to raise awareness of the diagnosis of *Histoplasma capsulatum* infection in both areas. From its discovery in the United States, a century ago to now its spread around the world, there are three main reasons: convenient travel and enhanced connectivity increases imported and exported cases of histoplasmosis; climate change and anthropogenic land utilization creates conditions that are more conducive to fungi (Azar et al., 2020); and the HIV pandemic and the widely use of immunosuppressive agents results in more cases of histoplasmosis (Wheat et al., 2016). Our case report and literature reviews are of great value in the context of a worldwide epidemic. Furthermore, after retrieving data of literatures from five databases, we found that the HLH with histoplasmosis in immunocompetent patient is extremely rare but had high mortality. Therefore, it is necessary to for clinicians to understand and recognize the disease.

Given what we already know, histoplasmosis endemic is highly associated with AIDS (Colombo et al., 2011; Rangwala et al., 2012; Nacher et al., 2013; Pan et al., 2013; Benedict et al., 2016). Our initial search results are consistent with this conclusion, a half of histoplasmosis infection cases were patients with HIV infection. An analysis on the United States reveals that the frequency of HIV-associated histoplasmosis hospitalizations has been decreased in the highly active antiretroviral therapy (HAART) era. In contrast, significant increases were observed for none of comorbidities and HIV seronegative immunosuppressed-associated hospitalizations (Benedict et al., 2016). Our literature search results are consistent with this trend.

A clear trigger usually was identified in most patients who recognized with acquired HLH. A retrospective multicenter study revealed that infectious trigger for the pathogenesis of HLH attribute to 33% adult patients (Schram et al., 2016). Common infectious agents incorporate EBV among viral infection as the most commonly implicated, Rickettsia and Mycobacterium among the bacteria, histoplasma among fungal pathogen, Plasmodium, Leishmania, and Babesia among parasites (Al-Samkari and Berliner, 2018).

The HLH-2004 criteria are used for diagnosis. Family history or molecular diagnosis is accord with HLH, or five of these eight criteria must be present (fever, splenomegaly, bicytopenia, hypertriglyceridemia and/or hypofibrinogenemia, hemophagocytosis, low/absent NK cell activity, hyperferritinemia, and high-soluble interleukin-2 receptor levels) (Henter et al., 2007). In our review of the literature, 12 patients with disseminated histoplasmosis meet the above five of the eight criteria, but one patient with insufficient information only fulfilled three criteria, and the definition of HLH was also given in the literature. NK cell activity were detected in only three cases, and high-soluble interleukin-2 receptor (CD25) were detected in half cases, as these tests might not be available in many medical centers (Ramos-Casals et al., 2014).

The standard diagnosis methods of histoplasmosis include growth of mold in culture and confirmation of yeast on cytopathology or histopathology of clinical specimens. Histoplasmosis mold form requires 6 weeks to grow in culture and may lead to delays in patient treatment. By contrast, histopathology and cytology have faster diagnosis process but lower sensitivity and specificity (Azar et al., 2020). Antigen detection represents a valuable diagnostic tool and a useful measure of treatment response (Swartzentruber et al., 2009; Hage et al., 2011). Serum antibodies have limited diagnosis utility for produced 4 to 8 weeks after acute histoplasmosis infection (Azar et al., 2020) and can yield false-negative and false-positive results (Gil-Brusola1 et al., 2007). Molecular diagnosis is used for the identification of suspected histoplasma capsulatum isolated from culture, and it characterized by rapid turnaround times, less susceptibility to host factors, and high sensitivity and specificity (Azar et al., 2020).

In our review, histological and cytological examination of all patients showed early indication of *H. capsulatum*; the fungus was further confirmed in bone marrow or blood culture in four studies. *H. capsulatum* was recognized by molecular diagnosis in two cases. Antigen and serum antibodies detection were used less common. As evidenced by the literature review presented here, the standard diagnosis methods also have valuable utility in immunocompetent patients.

HLH is a disorder of unchecked immune activation, and the main goal of treatment is to control the immune response that includes immunosuppressive and myelosuppressive agents. Specific treatment for HLH usually requires chemotherapy or HSCT accord to HLH-94 protocol, as is more common in familial HLH. In the cases reviewed, two patients were treated with dexamethasone and etoposide (DE); two patients were treated with Liposome doxorubicin, etoposide, and methylprednisolone (DEP). Three patients were initiated with intravenous immunoglobulin.

It is worth noting that 11 patients experienced clinical improvement and survived. Two of these patients had been deteriorating and died. Previous literature reviews suggest that the mortality rate of HIV-infected patients with HLH caused by disseminated histoplasmosis is 10%–44% (Gil-Brusola1 et al., 2007; Subedee and Van Sickels, 2015; Nguyen et al., 2020). Our result reveals similar prognosis of 15% mortality in immunocompetent patients, although our sample size is small.

Familial HLH is typically determined by genetic defect in immune function; most patients are infants under 2 years of age, but research has revealed that about 15% adult patients harbor mutations in familial HLH genes (Feldmann et al., 2002). *H. capsulatum* infections as a trigger for secondary HLH are primarily consider in our study, but exon detection of HLH mutated genes, NK cell activity, soluble interleukin-2 receptor (sIL2R), and interferon (IFN)-γ should be tested to support our speculation. Unfortunately, we did not verify it further. The presence of other hidden immunodeficiency disorders is also uncertain.

In summary, we provide a case report and literature review on hemophagocytic lymphohistiocytosis with histoplasmosis in immunocompetent patients, demonstrating the clinical features, diagnostic methods, and therapeutic outcomes. To the best of our knowledge, this is the first review in intact immunology patients. Prior analyses of HLH with histoplasmosis have been limited to a single immunocompetent case or to a specific patient population with AIDS (Subedee and Van Sickels, 2015; Nguyen et al., 2020). However, the number of cases in the literature that we were able to retrieve is limited. A significant proportion of patients may remain unreported or have been underdiagnosed. In view of the global epidemic trend and the improvement of fungal environment, it is necessary for clinicians to raise awareness of the incidence of this fungus in the general population.

## Data Availability Statement

The original contributions presented in the study are included in the article/**Supplementary Material**. Further inquiries can be directed to the corresponding author.

## Ethics Statement

Written informed consent was obtained from the individual for the publication of any potentially identifiable images or data included in this article.

## Author Contributions

HC, HH, and JW contributed to collection and collation the clinical data. QYu, HH, and HC contributed to extract data from literature and manuscript writing. TQ, MY, and QYa contributed to manuscript revisions and approved the final manuscript. All authors contributed to the article and approved the submitted version.

## Conflict of Interest

The authors declare that the research was conducted in the absence of any commercial or financial relationships that could be construed as a potential conflict of interest.

## Publisher’s Note

All claims expressed in this article are solely those of the authors and do not necessarily represent those of their affiliated organizations, or those of the publisher, the editors and the reviewers. Any product that may be evaluated in this article, or claim that may be made by its manufacturer, is not guaranteed or endorsed by the publisher.
